# Transformation of meta-stable calcium silicate hydrates to tobermorite: reaction kinetics and molecular structure from XRD and NMR spectroscopy

**DOI:** 10.1186/1467-4866-10-1

**Published:** 2009-01-14

**Authors:** Jacqueline R Houston, Robert S Maxwell, Susan A Carroll

**Affiliations:** 1 Chemistry, Materials, Earth, and Life Sciences Directorate; Lawrence Livermore National Laboratory, Livermore, CA 94550, USA

## Abstract

Understanding the integrity of well-bore systems that are lined with Portland-based cements is critical to the successful storage of sequestered CO_2 _in gas and oil reservoirs. As a first step, we investigate reaction rates and mechanistic pathways for cement mineral growth in the absence of CO_2 _by coupling water chemistry with XRD and NMR spectroscopic data. We find that semi-crystalline calcium (alumino-)silicate hydrate (Al-CSH) forms as a precursor solid to the cement mineral tobermorite. Rate constants for tobermorite growth were found to be *k *= 0.6 (± 0.1) × 10^-5 ^s^-1 ^for a solution:solid of 10:1 and 1.6 (± 0.8) × 10^-4 ^s^-1 ^for a solution:solid of 5:1 (batch mode; T = 150°C). This data indicates that reaction rates for tobermorite growth are faster when the solution volume is reduced by half, suggesting that rates are dependent on solution saturation and that the Gibbs free energy is the reaction driver. However, calculated solution saturation indexes for Al-CSH and tobermorite differ by less than one log unit, which is within the measured uncertainty. Based on this data, we consider both heterogeneous nucleation as the thermodynamic driver and internal restructuring as possible mechanistic pathways for growth. We also use NMR spectroscopy to characterize the site symmetry and bonding environment of Al and Si in a reacted tobermorite sample. We find two ^[4]^Al coordination structures at *δ*_*iso *_= 59.9 ppm and 66.3 ppm with quadrupolar product parameters (P_Q_) of 0.21 MHz and 0.10 MHz (± 0.08) from ^27^Al 3Q-MAS NMR and speculate on the Al occupancy of framework sites by probing the protonation environment of Al metal centers using ^27^Al{^1^H}CP-MAS NMR.

## Background

Burning of fossil fuels is believed to be the largest contributor to anthropogenic CO_2 _emissions and global climate change [1,2]. To reduce emissions and subsequently offset global warming, one solution is to inject CO_2 _into well-bores of depleted oil and gas reservoirs. Well- bores, however, are lined and plugged with Portland-based cement, which can chemically degrade in the presence of CO_2 _and water over time [3,4]. This presents a problem for long-term CO_2 _storage if reservoirs have the potential to leak through abandoned well sites. Deleterious effects can occur from leakage, including contamination of groundwater and subsurface resources and drastic changes to ecosystems [5-8]. In order to predict these processes and subsequently assess the long-term fate and storage of CO_2_, we need experimental data coupled with accurate simulations to identify reaction rates and pathways for cement dissolution and growth. However, there are few rate data on precipitation reactions and even fewer studies that derive growth mechanisms for cement-based minerals.

Calcium silicate hydrates are key components in cement minerals and have been suggested as precursor solids for the growth of stable minerals such as tobermorite and gyrolite [9,10]. Calcium silicate hydrates include many meta-stable and amorphous disordered structures, from which stable and highly crystalline materials such as tobermorite can form when heated. The mineral tobermorite is stable over a temperature range of ~80°C to ~150°C but can be produced at temperatures greater than 200°C as a meta-stable solid [9]. Orthorhombic tobermorite can be found as either a 9 Å, 11 Å or 14 Å polytype depending on the number of water molecules present in the structure. The structure of 11 Å tobermorite consists of layers of hydrated calcium ions bonded to repeating silicate chains that have bridging and non-bridging Si (Q^2^) and branching Si (Q^3^) sites [10-13]. The silicate chains repeat every third tetrahedron, giving rise to the terminology 'dreierketten' repeat. The Q^n ^notation often used to describe the silicate bonding represents the tetrahedron while the superscript refers to the number of other tetrahedra to which it is linked. When Al is available for reaction, Al substitution for Si in chain linkages can occur during growth. This is important to quantify because Al can affect growth rates [9], sorption properties [14], and the stabilities of cements [9]. While structural studies of Al-containing CSH and tobermorite phases are numerous [15-20], only a few studies have attempted to quantify rates and identify reaction mechanisms for tobermorite growth [9,10,21].

As a first step, we investigate the growth kinetics of tobermorite from a meta-stable calcium silicate hydrate in the absence of CO_2 _and suggest two possible reaction pathways for growth. We also use NMR to assign Al and Si coordination structures in a reacted sample of tobermorite and speculate on the Al occupancy of framework sites.

## Methods

### Batch-precipitation Experiments

Batch experiments were conducted at T = 150°C (± 1°C) and solution:solid ratios of 10:1 and 5:1. Amorphous silica (1.042 g; Mallinckrodt silicar: 306 m^2^/g surface area by BET, 75–100 *μ*m particle size), amorphous aluminum oxide (0.150 g; prepared by gibbsite calcination at 500°C for 5 h) and calcium oxide (0.940 g; prepared by calcite calcination at 1100°C for 6 h) solids were suspended in a 0.56 M NaOH to give stoichiometric ratios of Ca/(Al+Si) = 0.83 and Al/(Al+Si) = 0.15 [22,23]. The suspensions were mixed and transferred to Parr autoclave reactors and heated to T = 150°C (± 1°C). After heating for a specific amount time, reaction mixtures were quickly quenched within 30 min. A small amount of sample (~0.5 mL) was collected for pH measurement using an electrode that had been calibrated with standard buffer solutions (7.00, and 10.0 Fisher Scientific) at 25°C. The remaining solution (>10 mL) was then filtered through a 0.2 *μ*m membrane filter and acidified with 1 N HCl for Al, Si, and Ca analysis by inductively coupled plasma-atomic emission spectroscopy (ICP-AES). The reacted solid consisted of two distinct layered phases at early reaction times. All solids were crushed, filtered and washed three times with distilled water to remove residual ions and dried at 50°C overnight for X-ray powder diffraction and NMR analysis. No changes in pH were measured because the reaction mixture was buffered at pH ~13.3 by the sodic medium.

### Geochemical Calculations

Solution speciation, pH, and the saturation index for Al-substituted tobermorite were calculated at T = 150°C using the measured solution compositions from ICP-AES analysis at room temperature using the Yucca Mountain Project thermodynamic database (EQ3/6 code) [24]. This database was chosen because it contains a large amount of thermodynamic data for cement phases. The saturation indexes were calculated for both Al-CSH and tobermorite in which the saturation index is defined as *SI = Q/K*, where *K *is the solubility constant and *Q *is the activity quotient. The solubility constant for tobermorite was determined from the water composition for t = 4.5–8 d samples (*log K *= 52 (± 2)) and the solubility constant for Al-CSH was averaged over t = 15–24 h (*log K *= 44 (± 2)), where growth is at a maximum based on XRD (See *Equations 5 and 6*). All calculations were charge balanced on Na. The B-dot equation was used for the aqueous species activity coefficient model.

### X-ray Analysis

Powder diffraction profiles were obtained using a Siemens X-ray Diffractometer and APD3720 Philips Automatic Powder Diffractometer using CuK_*α *_radiation. For qualitative analysis, quick scans were obtained from 6–60 2*θ *using a 2s dwell time and 0.05 step.

Quantitative results were obtained by normalizing reflection intensities from tobermorite and the CSH gel to a known standard, *α*-Al_2_O_3 _(35.3 2*θ *reflection), using a longer dwell time and smaller scan range [23]. To determine the relative uncertainty, reflections were fit using three line-fitting routines; a pseudo-Voigt function with cubic-spline correction and K*α*_2 _fitting (JADE V.7.0), Pearson function, and a sum of Gaussian curves. Stoichiometry of the CSH gel and tobermorite solids was obtained from x-ray fluorescence analysis.

### NMR Spectroscopy

^27^Al MAS NMR spectra were collected on a Bruker Avance 400 wide-bore spectrometer (9.4 T) operating *ν*_o _= 104.25 MHz using a 4 mm triple-resonance solids probe. All sampleswere packed in zirconia rotors and spun at *υ*_*r *_= 12 kHz. Short single-pulse excitation times of 0.2 *μ*s (selective 90° = 1.7 *μ*s; 0.2 *μ*s ~ 10° tip angle) were used to yield near-quantitative ^27^Al signal intensities. Spectra were collected with 2 k data points, 0.5 s delay times, and averaged over 45,000 scans. All frequencies were referenced externally to a 0.1 M AlCl_3 _solution (*δ *= 0 ppm). Line-fit analyses were performed by fitting both tetrahedral and octahedral sites to two overlapping mixed Lorentzian-Gaussian functions. This routine was used only to integrate signal area. The standard deviations of the fitting parameters were less than 2%. Percent tetrahedral Al (^[4]^Al) and octahedral Al (^[6]^Al) were calculated by dividing the specific ^27^Al site area by the total observable ^27^Al NMR signal from the line-fit analysis (i.e. %^[4]^Al = (^[4]^Al/Al_total_) × 100) (Table 1). It is important to note that Al site percentages may not necessarily represent total Al due to the potential existence of unobservable Al sites with large quadrupolar interactions [25].

**Table 1:** Line-fits of NMR data to mixed Lorentzian-Gaussian functions show the gradual increase in ^[4]^Al with time (solution:solid of 10:1 batch reaction). Line-fit errors are < 2%.

time (h)	Total raw area counts for both ^[4]^Al sites (arbitrary units)	%^[4]^Al = ^[4]^Al/Al_total _× 100
0	7.16 × 10^4^	20
3	8.30 × 10^4^	30
6	12.0 × 10^4^	51
9	15.5 × 10^4^	57
15	20.3 × 10^4^	68
19	21.5 × 10^4^	81
24	25.2 × 10^4^	86
30	30.4 × 10^4^	97
42	29.4 × 10^4^	95
48	32.1 × 10^4^	95
72	30.8 × 10^4^	100
120	31.9 × 10^4^	100

The optimum match condition for the ^27^Al{^1^H}CP-MAS NMR experiment was setup on Al(OH)_3_. Spectra were obtained using a 4 *μ*s ^1^H pulse and 1 s delay. A total of 1 k data points were collected over 55,000 scans. Short contact times of 300 *μ*s were used to avoid selectively exciting one Al site over the other and to minimize signal loss due to short Al *T*_*1ρ *_relaxation times [26,27]. Typical selective ^27^Al 90° pulse widths (*pw*) were 1.7 *μ*s. ^27^Al triple-quantum MAS (3Q-MAS) NMR spectra were obtained using a 3-pulse sequence with z-filter [28]. For the 3-pulse sequence, an excitation pulse of 5.5 *μ*s, conversion pulse of 2.2 *μ*s, followed by a weak selective 90° pulse of 30 *μ*s was used with rotor synchronization [29]. A total of 32 points in the *t*_*1 *_dimension were collected with 20 ms increments, each corresponding to 1860 acquisitions. Spectra were acquired using a 1s recycle time. The data were processed using a shear transformation in the indirect dimension with line-broadening of 100 Hz [30]. Full simulations of 3Q-MAS slices were not performed due to considerable overlap of the ^[4]^Al signals. Isotropic chemical shifts and quadrupolar product parameters (P_Q_) were calculated for the t = 24 h sample (solution:solid 5:1). The quadrupolar product parameter is defined as $P_{Q}=C_{Q}\sqrt{1+(n_{Q}^{2}/3)}$ and was calculated by running the sample at two magnetic field strengths (9.4 T and 11.7 T) to determine the field dependence of the central transition for both ^[4]^Al sites [31].

^29^Si MAS NMR spectra were collected using a 300 MHz Tecmag-Apollo spectrometer operating at *ν*_*o *_= 59.64 MHz. A 7.5 mm double-resonance Chemagnetics probe was used and all samples were spun at 3.0(± 0.5) kHz. A 5 *μ*s pulse and 4 sec delay were used to collect ^29^Si MAS NMR spectra for approximately 2 days (3000 scans). A total of 512 data points were collected but zero-filled to 1 k during data-processing. All chemical shifts are referenced externally to TMS and spectral intensities were normalized by the dry weight of the sample.

## Results and Discussion

### Reaction of CaO/Al_2_O_3_/SiO_2 _in alkaline solution

We find that reaction of CaO/SiO_2_/Al_2_O_3 _in alkaline solution produces CSH (amorphous CSH and semi-crystalline Al-CSH) and semi- and fully-crystalline tobermorite at T = 150°C (solution:solid 10:1, 5:1). These reactions are shown in *Equation 1 *and supported by spectroscopic and diffraction data that is described in detail in the following sections.

(1) CaO + SiO_2 _+ Al_2_O_3 _→ CSH_(amph)_/Al-CSH_(s-cryst) _→ Tobermorite_(s-cryst) _→ Tobermorite_(cryst)_

**Figure 1:**
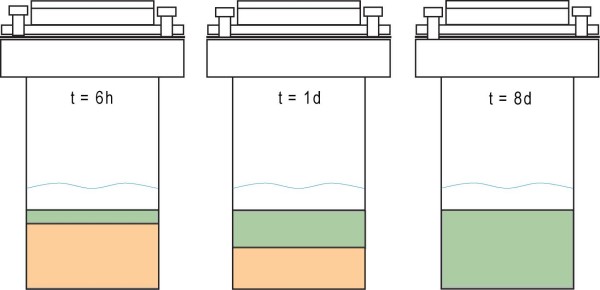
**Schematic representation of static batch reactions for t = 6 h, 1 day, and 8 days (10:1 batch mixture)**. At early reaction times, the solid cake consists of two distinct layers in which the top layer is tobermorite (green) and the bottom is unreacted Al-CSH gel (orange). Water is shown in blue. These diagrams illustrate that the amount of tobermorite increases with reaction time and is complete after 8 days of heating.

**Figure 2:**
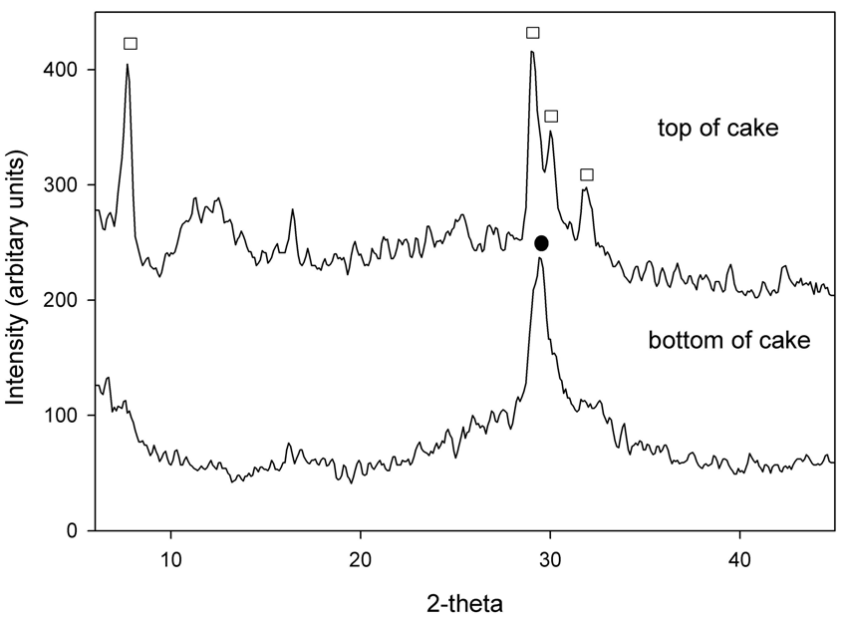
**Powder diffraction data of a physically separated solid cake**. The top layer shows reflections at 7.8 2*θ *(002), 29.1 2*θ *(220), and 30.1 2*θ *(222) that are indicative of 11 Å tobermorite (White Square) while the bottom layer shows one broad reflection at 29.5 2*θ *due to CSH (Black Circle).

All experiments were run in batch mode in which the solution composition was sampled for water chemistry and solid phases were analyzed by XRD and NMR spectroscopy (Table 2 for water chemistry data). Due to the static nature of the batch experiments, most reacted solids consisted of two phases in which a hard white crust was layered upon a soft gel. Comparison of several batches show that the thickness of the top layer increased while the bottom gel layer decreased with prolonged periods of heating time (Figure 1). When the two layers of the solid were physically separated and analyzed by XRD, the top layer showed reflections at 7.8 2*θ *(002), 29.1 2*θ *(220), 30.1 2*θ *(222) indicative of 11 Å tobermorite and the bottom layer showed one broad reflection at 29.5 2*θ *due to CSH (Figure 2). No other crystalline phases were detected in any diffraction patterns collected in this study. XRF analyses of CSH and tobermorite samples were performed to determine the stoichiometries for both solid phases. An Al-CSH gel separated from a reaction mixture that was heated for 21 h (10:1 solution:solid) was found to have stoichiometric ratios of (Ca+Na)/(Al+Si) = 0.88 and Al/(Si+Al) = 0.18. A tobermorite sample heated for 8 days was found to have stoichiometric ratios of (Ca+Na)/(Al+Si) = 0.92 and Al/(Si+Al) = 0.14. This gives calculated chemical formulas of Ca_3.3_Na_2.1_Si_5.1_Al_1.1_O_16_(OH)_2 _• 6.2H_2_O for Al-CSH and Ca_4.3_Na_1.4_Si_5.3_Al_0.9_O_16_(OH)_2 _• 4.9H_2_O for tobermorite, assuming that the O and OH compositions have ideal stoichiometry. This data indicates that growth of tobermorite occurs at the top of the gel phase and that these phases have similar stoichiometries after 1 day of reaction.

**Table 2:** Aqueous silica, aluminum and calcium concentrations from the 10:1 batch reaction.

time (h)	Si_(aq)_ (mol kg^-1^)	Al_(aq)_ (mol kg^-1^)	Ca_(aq)_ (mol kg^-1^)
0.0	0.00165	0.00021	0.00182
3.0	0.00934	0.00050	0.00002
6.0	0.00501	0.00101	0.00002
9.0	0.00312	0.00127	0.00006
15.2	0.00126	0.00140	0.00010
19.0	0.00038	0.00136	0.00021
48.0	0.00154	0.00279	0.00008
24.0	0.00123	0.00146	0.00021
42.0	0.00303	0.00186	0.00005
30.6	0.00057	0.00227	0.00018
24.0	0.00062	0.00186	0.00024
72.0	0.00674	0.00188	0.00004
120.0	0.00286	0.00163	0.00006
47.8	0.00215	0.00257	0.00006
94.5	0.00250	0.00062	0.00006
48.0	0.00163	0.00355	0.00006
190.5	0.00390	0.00034	0.00002
96.5	0.00313	0.00100	0.00003
64.0	0.00823	0.00362	0.00005
89.0	0.00695	0.00201	0.00006
16.0	0.00100	0.00101	0.00010

Standard deviations are ± 4–5 × 10^-4 ^mol kg^-1 ^for Si_(aq) _and Al_(aq) _and at most ± 9 × 10^-5 ^mol kg^-1 ^for Ca_(aq)_.

### Molecular structure of tobermorite by MAS NMR

**Figure 3:**
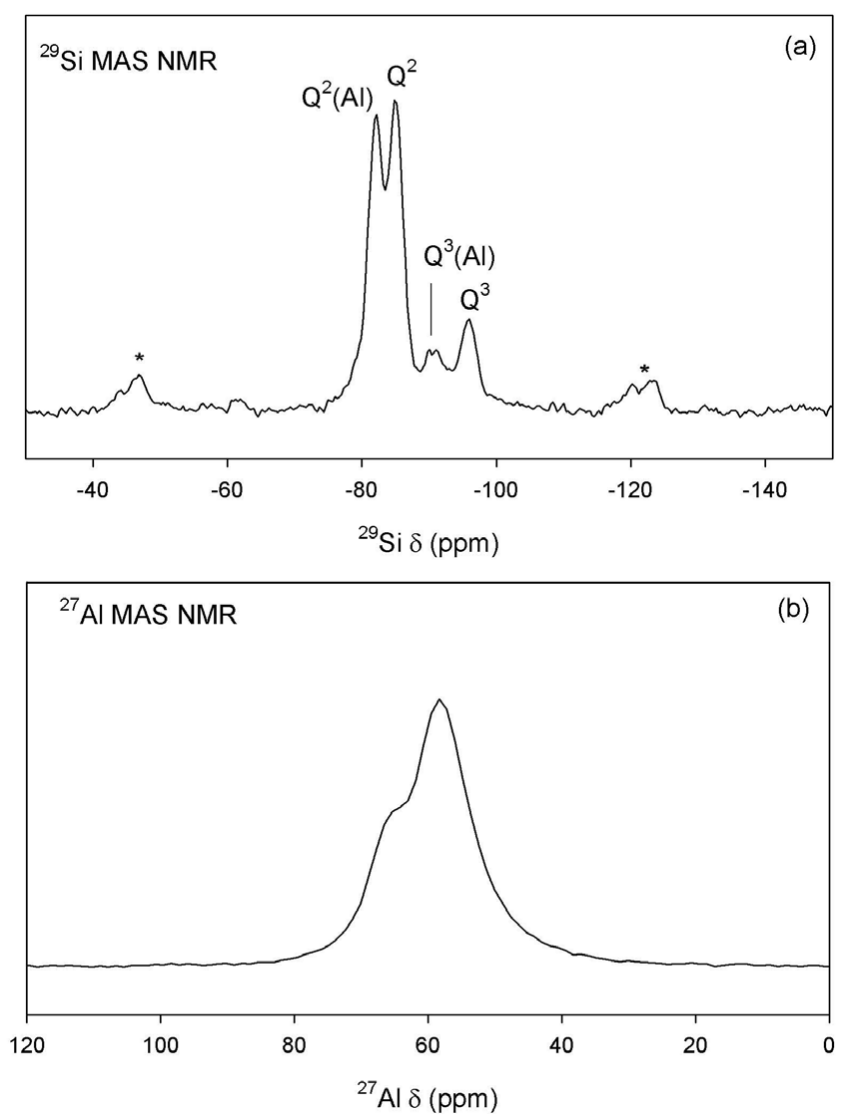
**^29^Si MAS NMR (a) and ^27^Al MAS NMR (b) of tobermorite that had been reacted for t = 24 h (solution:solid 5:1)**. ^29^Si MAS NMR shows Q^2 ^and Q^3 ^Si-O-Si and Si-O-Al chain linkages while ^27^Al MAS NMR shows two structurally distinct ^[4]^Al coordination sites from Al in bridging and branching bonds.

NMR spectroscopy was used to characterize the site symmetry and bonding environment of Al and Si in tobermorite. Shown in Figures 3a and 3b are the ^29^Si MAS and ^27^Al MAS NMR data for a reacted sample that contains tobermorite and a small amount of semi-crystalline Al-CSH (5:1 batch reaction; t = 24 h; See diffraction data; Figure Eleven). We observe from ^29^Si MAS NMR two signals at *δ *= -81 ppm and -85 ppm due to bridging and non-bridging Q^2 ^Si-O-Al and Si-O-Si linkages, respectively. We also observe two distinct signals at *δ *= -92 ppm and -96 ppm due to branching Q^3 ^Si-O-Al and Si-O-Si bonds, respectively (Figure 3a). Branching Q^3 ^signals have lower signal intensity because there are fewer Q^3 ^sites that link across interlayers compared to Q^2 ^chain sites (ideal composition: 1:2 for tobermorites with Ca/Si ratios near 0.83) [32]. These data show that tobermorite consists of Si-O-Al chain units and that there is cross-linking, as expected, for a tobermorite with a low Ca/(Si+Al) ratio [33].

**Figure 4:**
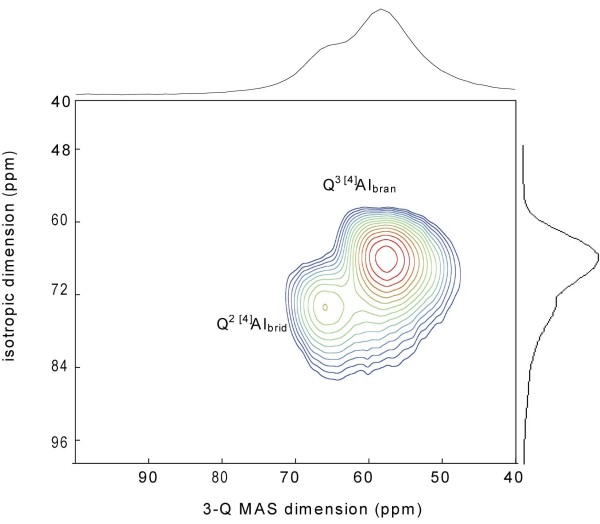
**^27^Al 3Q-MAS NMR of tobermorite (t = 24 h sample)**. These data show two structurally distinct ^[4]^Al sites from Q^2 ^bridging (Q^2 [4]^Al_brid_) and Q^3 ^branching (^3^Q ^[4]^Al_bran_) chains within the tobermorite framework.

^27^Al MAS NMR was used to identify Al coordination structures based on chemical shifts (^[4]^Al *δ *= ~50–70 ppm, ^[5]^Al *δ *= ~30–40, ^[6]^Al *δ *= ~0 ppm). Our NMR data show at least two distinct ^[4]^Al coordination sites with centerbands at ~58 ppm and 65 ppm (Figure 3b), which we resolve using ^27^Al 3Q-MAS NMR spectroscopy (Figure 4). The 3Q-MAS NMR method is a two-dimensional technique that removes the second-order quadrupolar contribution to the quadrupolar broadening for nuclei with spin>1/2 (i.e. ^27^Al). Thus, this technique allows us to resolve structurally similar but distinct ^[4]^Al coordination sites within the silicate framework. We find two ^[4]^Al coordination sites at *δ*_*iso *_= 59.9 ppm and 66.3 ppm with P_Q _= 0.21 MHz and 0.10 MHz (± 0.08), respectively, indicating a high degree of structural order within the tobermorite framework. These spectroscopic features and quadrupolar parameters are indicative of aluminous tobermorites and ^[4]^Al substituted calcium silicate hydrates [32-37].

**Figure 5:**
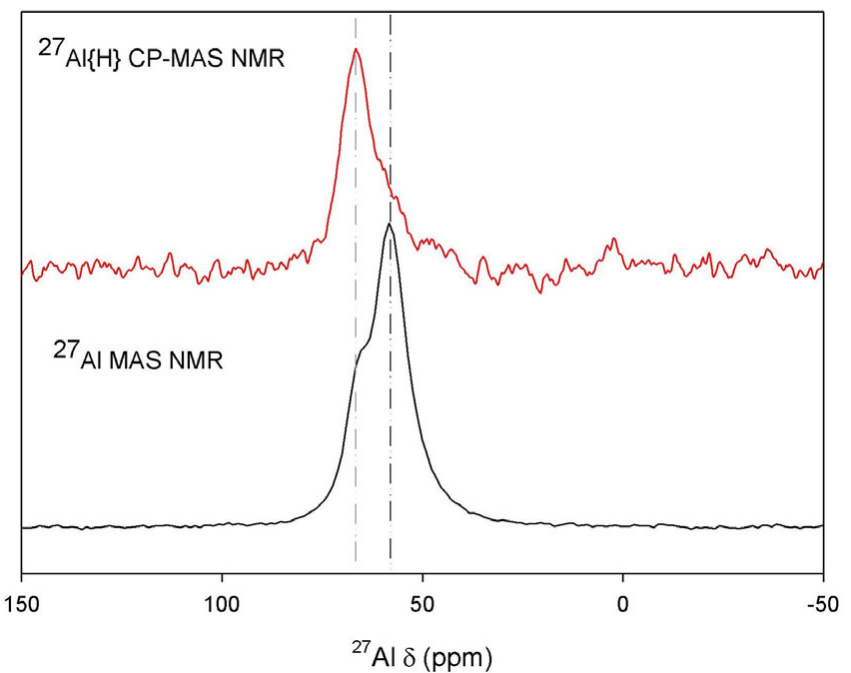
**^27^Al{^1^H}CP-MAS NMR of tobermorite (t = 24 h sample)**. These data show polarization transfer from both sites in which CP transfer is greater for the ^[4]^Al site at 65 ppm than for the site at 58 ppm (solution:solid of 5:1).

We show that growth of Al in tobermorite occurs in two distinct structural positions and suggest that these positions are Q^2 ^bridging and Q^3^branching coordination sites. These assignments have also been proposed by Sun et al. [33], Komarneni [32,34] and Gabrovsek et al [38] for Al tobermorites who suggest that Al occupies bridging and branching bonds. However, Faucon et al [37] propose that Al occupies non-bridging sites and that with increasing Ca concentrations, Al redistributes via an internal restructuring mechanism to occupy bridging bonds. We constrain these assignments by probing the protonation environment of Al metal centers using ^27^Al{^1^H}CP-MAS NMR because the relative protonation states of Q^2 ^bridging, Q^3 ^branching, and Q^2 ^non-bridging sites are likely to be different due to differences in the number of bound oxo bridges (bridging Q^2 ^= AlO_2_(OH)_2_; branching Q^3 ^= AlO_3_OH; non-bridging Q^2 ^= AlO_4_; assuming maximum protonation which is likely for tobermorites with low Ca/(Si+Al) ratios; See Ref [39]). ^27^Al{^1^H}CP-MAS NMR allows for the transfer of polarization from ^1^H to ^27^Al by exploiting heteronuclear dipolar couplings [40]. As such, we can use CP-MAS to identify ^27^Al coordination sites that are near OH or H_2_O molecules. CP-MAS data shows that both ^[4]^Al sites exhibit polarization transfer from nearby protons and that CP transfer is most significant for the ^[4]^Al site at 65 ppm, which is in lower abundance (Figure 5). CP-MAS data also show a small amount of CP transfer for the ^[4]^Al site at 58 ppm, however, signal in this spectral region may contain a contribution from the quadrupolar broadened resonance at 65 ppm. We anticipate that the contribution from the quadrupolar broadened resonance at 65 ppm is small though due to the structural order of the solid. Therefore, polarization transfer in this region is most likely from the 58 ppm ^[4]^Al site only. Based on this data, we propose that the 65 ppm signal is due to Q^2 ^^[4]^Al bridging sites, consistent with the structural model proposed by Richardson [39,41] who show Q^2 ^bridging sites with terminal OH bonds. We suggest that the signal at 58 ppm is due to Q^3 ^^[4]^Al branching sites because Q^3 ^sites are expected to contain fewer OH groups than Q^2 ^bridging units due to cross-linking across silicate layers. This is consistent with the low polarization transfer observed for this site. Had Al occupancy of non-bridging bonds taken place, we would expect no CP signal at 58 ppm because these sites contain only bridging oxo groups. We should note, however, that CP MAS does not give direct evidence of Al-OH sites, but detects protons that are near Al nuclei. Polarization transfer may be due to protons from intracrystalline water, in which the differences in polarization efficiency at the two Al sites could be due to the proximity of intracrystalline waters to metal centers. We also note that differences in polarization transfer for the two sites may be due to differences in relaxation times (T_1*ρ*_^Al^) as discussed by Morris and Ellis [42] (See also Ref. [26]). However, we chose short contact times to avoid the selective excitation of one Al site over the other and to minimize signal loss due to short Al T_1*ρ *_relaxation times. Although CP MAS does not allow us to conclusively identify Al-OH sites, several studies have shown that Al metal centers in tobermorites contain bound hydroxyls based on IR spectroscopy (Al-OH bands at 935-930 cm^-1^; Ref [43]). Based on this data, Al occupancy of Q^2 ^bridging and Q^3^branching sites suggests that Al links silicate polymeric chains together during the growth mechanism, creating silicate units that are connected by bridging and branching Al tetrahedra [44,45].

### Reaction mapping using NMR

**Figure 6:**
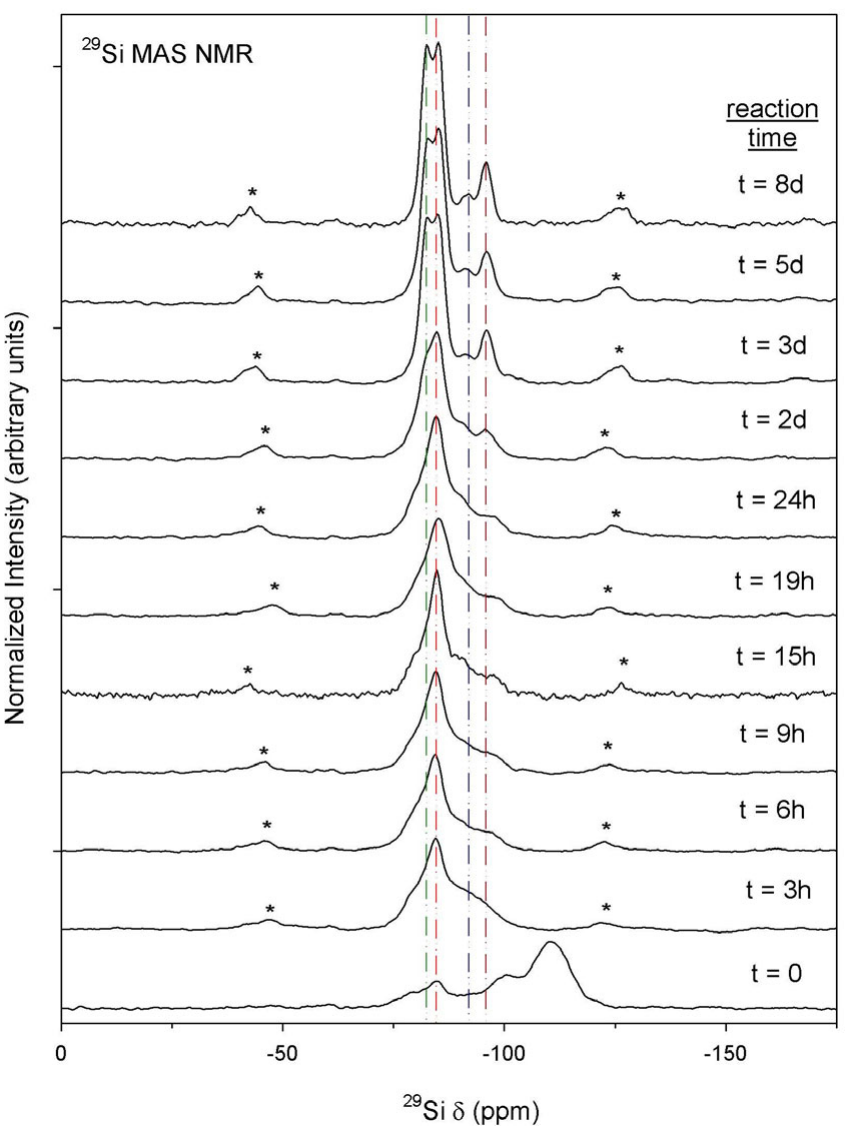
**^29^Si MAS NMR of batch reactions at a solution:solid ratio of 10:1**. Asterisks denote spinning side-bands. For the fully reacted sample (t = 8 d), the two signals at *δ *= -81 ppm and -85 ppm are due to bridging and non-bridging Q^2 ^Si-O-Al and Si-O-Si linkages, respectively. The two signals at *δ *= -92 ppm and -96 ppm are due to branching Q^3 ^Si-O-Al and Si-O-Si bonds, respectively.

**Figure 7:**
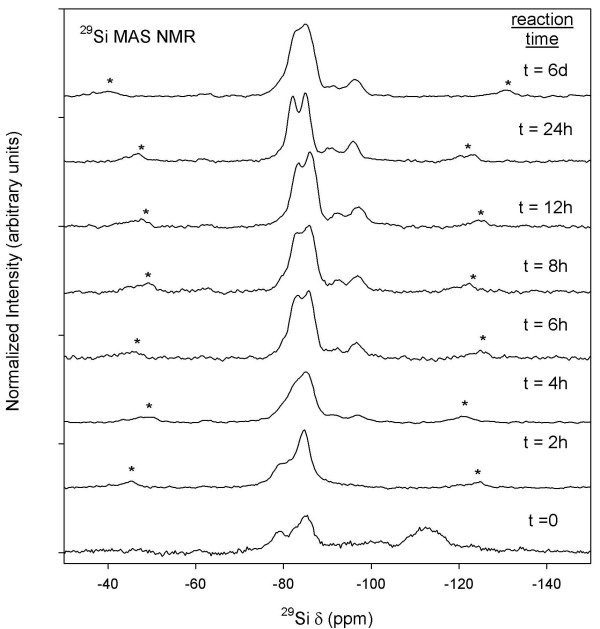
**^29^Si MAS NMR of batch reactions at a solution:solid of 5:1**. Asterisks denote spinning side-bands.

^29^Si MAS and ^27^Al MAS NMR spectroscopy was used to monitor the disappearance of starting materials and map the growth of Al-CSH and tobermorite. We show in Figure 6^29^Si MAS NMR spectra from the batch reaction at a solution:solid ratio of 10:1. At t = 0, we observe two broad signals at -101 and -110 ppm due to unreacted amorphous silica sites, which disappear in t < 3 h. We also observe a broad shoulder at -79 ppm and a signal at -85 ppm due to Q^1 ^and Q^2 ^Si-O-Si sites most likely from the hydrated CSH gel. At t >3 h we observe line-shapes at -82 and -85 ppm due to Q^2 ^Si-O-Al and Si-O-Si bonds, which are better resolved at longer reaction times due to increased crystallinity of the solid phase (See t = 2–3 d; Figure 6). We also observe the appearance of broad signals from -92 ppm to -96 ppm due to Q^3 ^Si sites with and without one Al next nearest neighbor, respectively [33,46]. Increased resolution of coordination sites indicate that tobermorite growth is nearly complete after 3d, and that re-crystallization of the solid phase occurs. Results from the batch reaction at a solution:solid ratio of 5:1 are shown in Figure 7 and are similar to those for the 10:1 batch reaction except that growth kinetics are much faster. We observe amorphous silica at t = 0, Q^1 ^and Q^2 ^Si sites from the gel at t = 2 h, followed by growth and crystallization of Q^2 ^and Q^3 ^Si-O-Al and Si-O-Si sites at longer reaction times. We also note, that at t = 6d the tobermorite structure looses some degree of crystallinity because the ^29^Si MAS NMR spectra show lower resolution for the Q^2 ^Si-O-Si and Si-O-Al bridging bonds at -82 and -85 ppm. This decrease in spectral resolution may indicate an increase in structural disorder at long reaction times that is not measureable by XRD (Refer to Figure Ten discussed in the following section).

**Figure 8:**
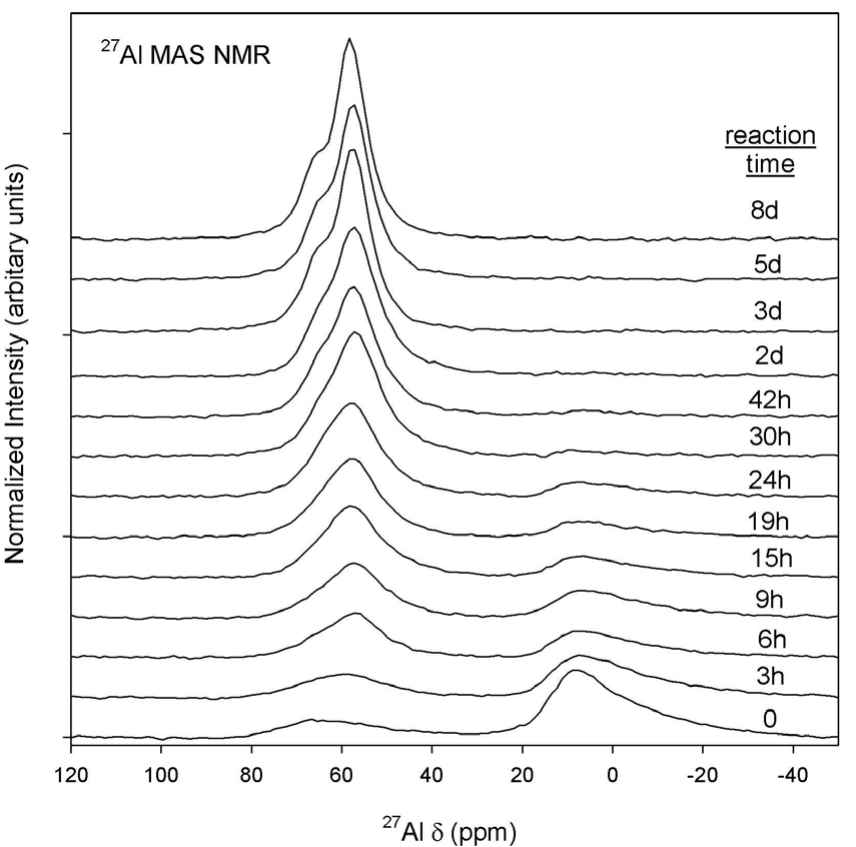
**^27^Al MAS NMR of amorphous Al_2_O_3 _starting material (^[6]^Al ~ 9 ppm ; ^[4]^Al ~ 62–70 ppm) and Al-CSH and tobermorite solids (^[4]^Al ~ 54–68 ppm) for the 10:1 batch reaction**.

**Figure 9:**
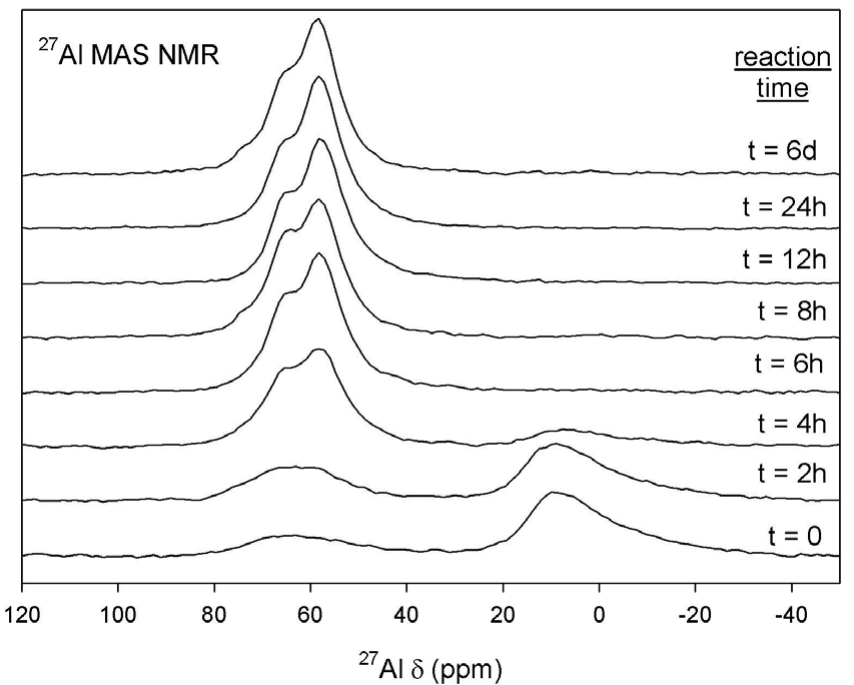
**^27^Al MAS NMR of amorphous Al_2_O_3 _starting material, Al-CSH and Al-incorporated tobermorite solids for solution:solid of 5:1 batch experiments**. Comparison of these data with Figure 8 show that reaction rates are faster when the solution volume is reduced by half.

^27^Al MAS NMR data show broad line-shapes at roughly ~9 ppm and ~62 ppm at t = 0 and 3 h (10:1 batch reaction; Figure 8). These signals are due to ^[6]^Al and ^[4]^Al, respectively, and are characteristic of Al coordination sites in amorphous Al_2_O_3 _starting material [47]. After t > 0, the ^[4]^Al site intensity increases with reaction time, indicating growth of Al-CSH and tobermorite phases that are indistinguishable by NMR. During t = 24 h–30 h, the ^[6]^Al signal due to Al_2_O_3 _starting material disappears and we observe two chemically distinct ^[4]^Al sites at ~58 ppm and 65 ppm. Reaction rates for growth of both ^[4]^Al coordination sites were difficult to determine, particularly for short reaction times where the ^[4]^Al sites are poorly resolved and residual Al-CSH and Al_2_O_3 _are still present. Qualitatively, however, the bridging site at 66 ppm appears to reach steady-state faster than the branching site at 58 ppm, although this is difficult to discern due to spectral overlap. Komarneni et al [32] show that at low Al levels, the ratio of the Q^3^/Q^2 ^site is much less than we show here (Q^3^/Q^2 ^site ~1/1; Figure 1c Ref [32]), suggesting that Q^2 ^^[4]^Al units reach steady-state faster while the growth of Q^3 ^^[4]^Al cross-linked units are limited by the amount of Al available for reaction. When the total area of ^[4]^Al sites are determined by fits to Lorentzian-Gaussian lines, we find that %^[4]^Al does not significantly increase after 3 days of reaction (%^[4]^Al = (^[4]^Al/Al_total_) × 100; Table 1). This suggests that tobermorite growth has reached near equilibrium and that increasing signal intensity and narrowing of line-shapes is due to re-crystallization, as previously suggested from ^29^Si MAS NMR. The ^27^Al MAS NMR data for batch reactions with a solution:solid ratio of 5:1 are virtually identical except that reaction rates are considerably faster (Figure 9). ^27^Al MAS NMR spectra show the disappearance of amorphous alumina starting material (t > 4 h), the growth of two ^[4]^Al sites at approximately 57 ppm and 64 ppm (t > 2 h), and structural crystallization of the tobermorite solid after t > 8 h. Two ^27^Al MAS NMR spectra (t = 8 h, 6 d) show a small signal at 72 ppm due to Al-O-Al bonds, most likely from residual amounts of starting material [47]. This data shows that reaction rates for tobermorite growth are considerably faster at lower solution volumes.

Although we are not able to quantify reaction kinetics using NMR data due to the lack of resolution and complicated nature of the spectra, NMR allows us to gain distinct structural information and to monitor the dissolution of amorphous starting materials that are undetectable by XRD. As we show in the following section, we use XRD as a compliment to determine bulk reaction rates for tobermorite growth.

### Reaction progress from XRD

**Figure 10:**
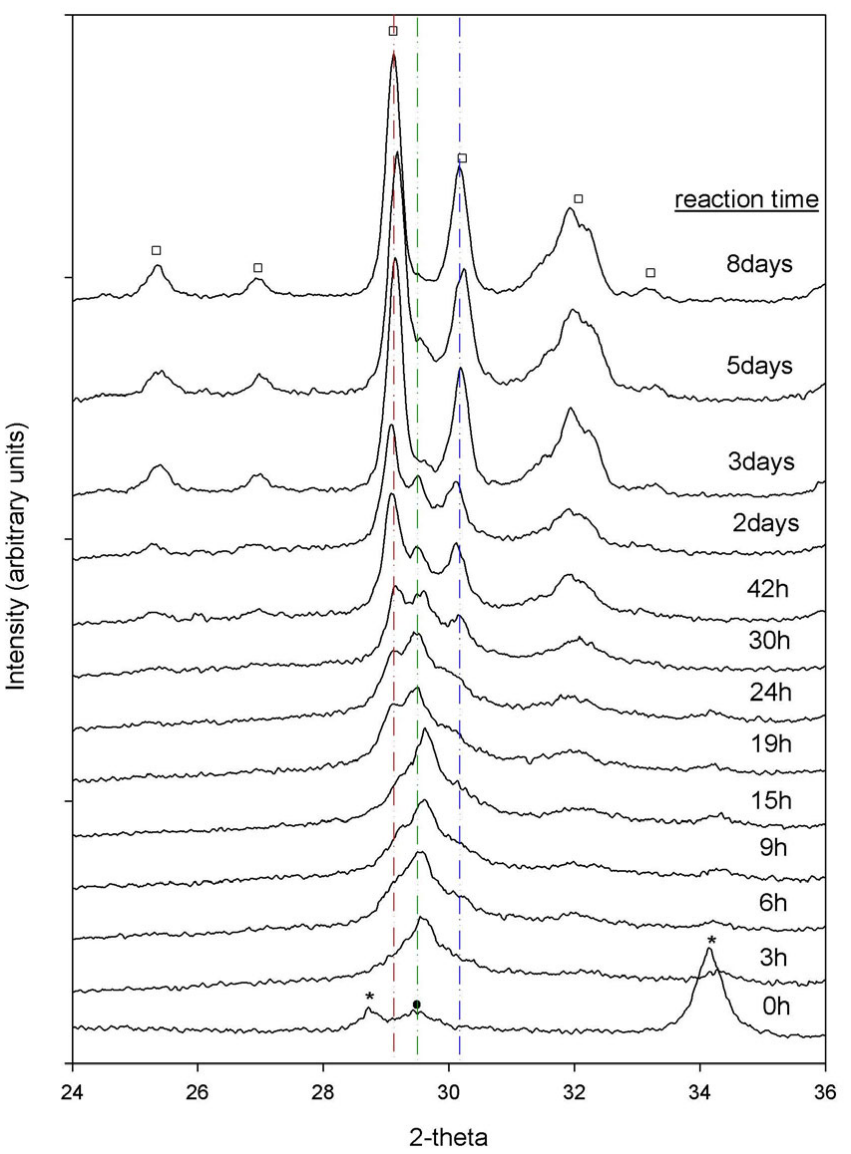
**X-ray powder diffraction profiles for the 10:1 batch reaction (T = 150°C)**. Powder patterns show the dissolution of portlandite(*), growth and disappearance of CSH (Black Circle), and growth and crystallization of 11Å tobermorite (White Square).

**Figure 11:**
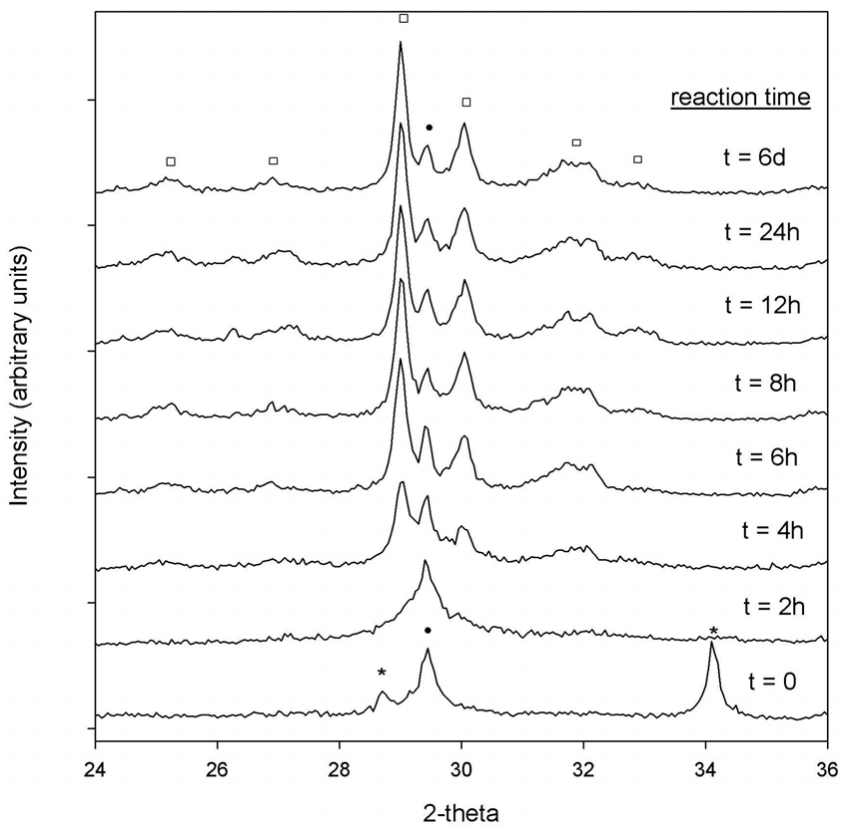
**X-ray diffraction patterns for the 5:1 batch reactions at T = 150°C**. Dissolution of portlandite(*), growth and disappearance of CSH (Black Circle), and growth and crystallization of 11 Å tobermorite (White Square) are observed from the powder patterns.

**Figure 12:**
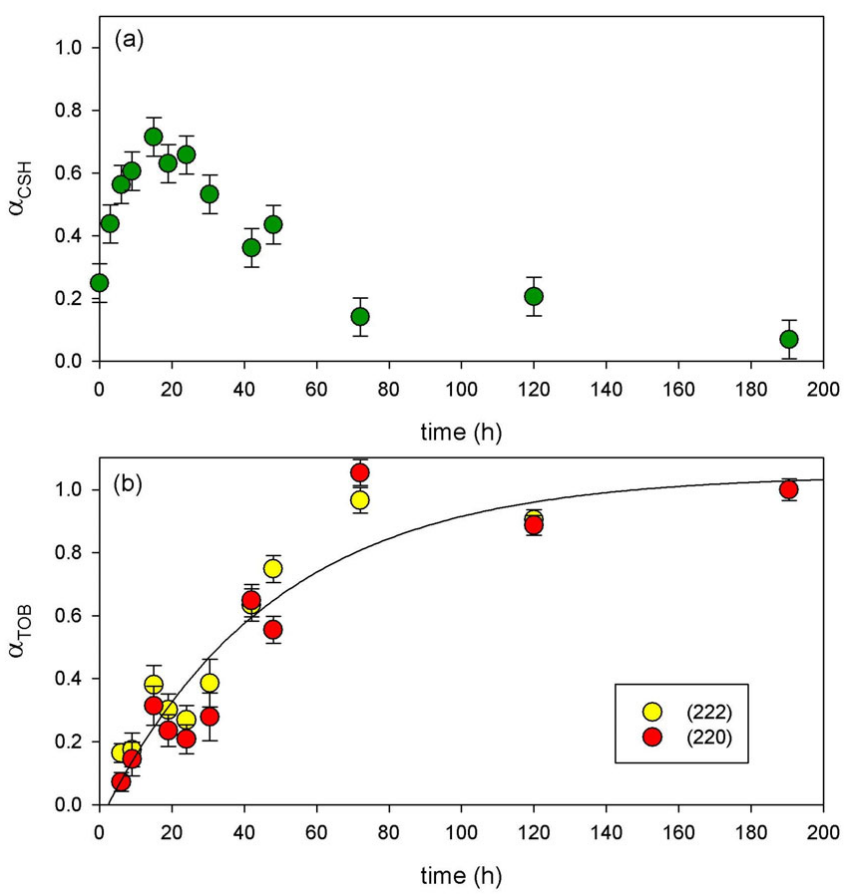
**Line-fits to the Al-CSH gel XRD reflection at 29.5 2*θ *(a) and tobermorite XRD peaks at 29.1 2*θ *(220) and 30.1 2*θ *(222) (b) are shown for the 10:1 batch reaction**. The data in (a) illustrate the growth of CSH until t ~19–24 h followed by consumption. The data in (b) show the growth of tobermorite (t < 2 d) followed by crystallization from t = 3–8 d. Line-fit performed using the Avrami equation (*Equation 4*) in which *k *= 0.6 (± 0.1) × 10^-5 ^s^-1 ^and *t*_*o *_= 2.3.

**Figure 13:**
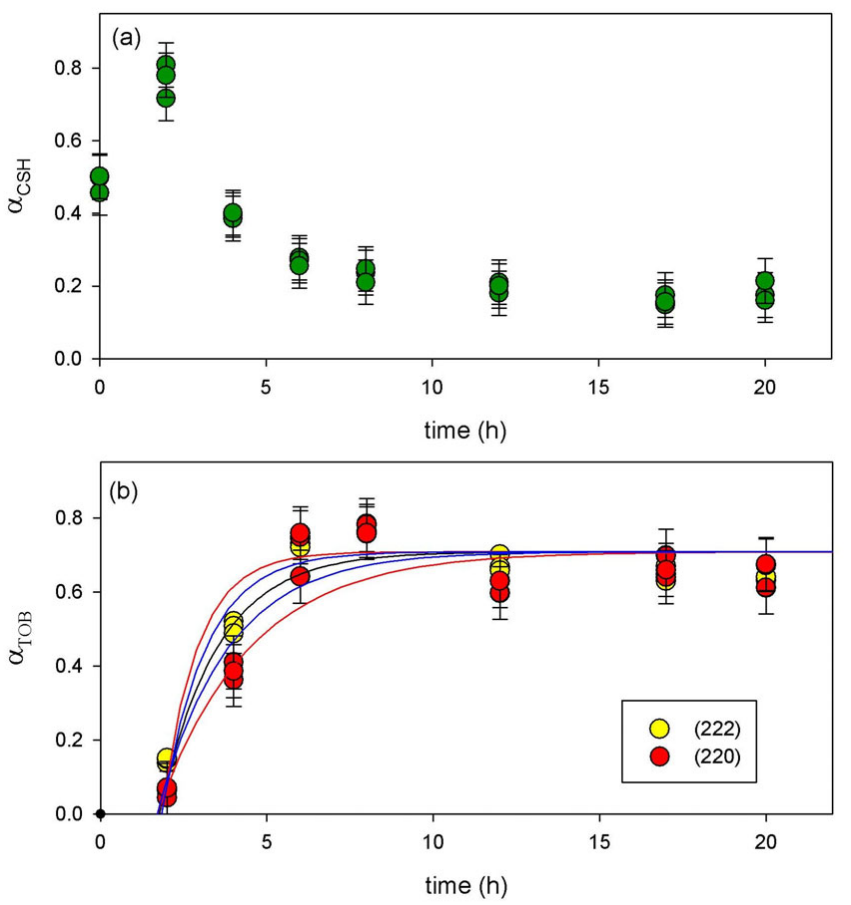
**Line-fits to the Al-CSH and tobermorite XRD reflections from the 5:1 batch experiments**. The data in (a) show the growth of semi-crystalline Al-CSH at early reaction times (t < 2 h) followed by consumption. The data in (b) show the growth of tobermorite followed by crystallization. Line-fit analysis of the tobermorite growth curve was performed using the Avrami equation (*Equation 4*) in which *k *= 1.6 (± 0.8) × 10^-4 ^s^-1 ^and *t*_*o *_= 1.7. Blue lines represent ± 0.4 × 10^-4 ^s^-1 ^and red lines represent ± 0.8 × 10^-4 ^s^-1 ^errors in the calculated rate constant.

Growth and disappearance of semi-crystalline Al-CSH and crystalline tobermorite were examined by monitoring changes in powder diffraction profiles. XRD data for the batch reactions at solution:solid ratios of 10:1 and 5:1 are shown in Figures 10 and 11 and plots of the extent of reaction are shown in Figures 12 and 13 (T = 150°C). Figure 10 for the 10:1 batch reaction shows that growth of tobermorite and disappearance of Al-CSH are relatively rapid in alkaline medium and are nearly complete after 3 days. At t = 0, we observe residual portlandite reflections, 28.7 and 34.2 2*θ*) and the onset of semi-crystalline Al-CSH at 29.5 2*θ *and 49.5 2-theta (49.5 2-theta signal not shown). Al-CSH continues to grow until t ~ 19–24 h, after which the reflection intensity decreases as the gel is consumed. Because the dissolution of Al_2_O_3 _starting material is somewhat slow (^[6]^Al persists up to 1 day on the ^27^Al MAS NMR spectra; See Figure 4), semi-crystalline Al-CSH likely has varying amounts of Al during the first 24 h of the growth reaction. During t = 3–6 h, we observe peaks at 29.1 2*θ *and 30.1 2*θ *that flank the CSH reflection due to 11 Å tobermorite, which continues to grow until approximately t = 3 d. Comparison of t = 3 d with t = 8 d shows a small increase in signal intensity and a slight narrowing of line-shapes, which suggests that re-crystallization of the tobermorite structure occurs rather than continued growth of the mineral phase. We confirm these observations by showing that the total signal area for ^[4]^Al sites on the ^27^Al MAS NMR spectra are relatively constant over the period of 3–8 days (Table 1). For the batch reactions with a solution:solid ratio of 5:1, we observe similar trends in the diffraction data except that reaction rates are significantly faster (Figure 11). We observe from the powder patterns, growth of semi-crystalline Al-CSH, consumption of Al-CSH until steady state is approached and growth of semi-crystalline and crystalline tobermorite over time. This data set shows that even after 6 days of reaction time, there is slightly more Al-CSH gel remaining in the mixture when compared to the 10:1 batch composition at t = 8 d.

We calculate the extent of reaction (*α*) by normalizing the area counts of Al-CSH and tobermorite reflections to maximum peak areas (*Equation 2*) and then assume the sum of the areas for the Al-CSH and tobermorite reflections are equal to 1 at long reaction times (*Equation 3*). XRD and NMR data show that this is a valid assumption because virtually no CaO/SiO_2_/Al_2_O_3 _starting material is left after 24 h for the 10:1 batch reaction and 6–8 h for 5:1 batch mixture.

(2) $$\alpha_{CSH,Tob}=\frac{{\left(area\right)}_{t}}{{\left(area\right)}_{\max}}$$

(3) *α*_*CSH *_+ *α*_*Tob *_= *1*

Comparison of Al-CSH and tobermorite data show that the summation of *α*_Tob _and *α*_CSH _is less than 1 during the first 15 h of reaction for the 10:1 batch experiment (Figures 12). This indicates the existence of an x-ray amorphous phase that is not directly detectable by XRD. Both ^29^Si NMR (Figures 6 and 7) and XRD (Figures 10 and 11) data show that the bulk of the starting material (silica gel and CaO) has dissolved during this time. Thus, this x-ray amorphous phase is most likely an amorphous Al-poor CSH. This is not surprising considering several studies have reported various CSH phases with different compositions and crystallinities (See Ref [20] and references therein). For example, Stade [48,49] proposed that there are three forms of CSH; tobermorite-like polymeric CSH, dimeric amorphous CSH and a mixture of both. However, we can not speculate on the growth or structure of amorphous CSH because we have no direct evidence from spectroscopy or XRD that this phase exists.

### Bulk reaction rates for tobermorite growth

To calculate a growth rate for tobermorite for both batch experiments, x-ray reflections at 29.1(222) and 30.1(220)2*θ *were normalized to an internal standard and then plotted as the extent of reaction (*α*) with respect to time (Figure 12 and Figure 13). Although there is considerable error in the fitting routine, line-fit analyses show that tobermorite growth is exponential and can be fit to the Avrami model [50-52], which is often used to describe solid-state reactions such as crystallization [53-55], crystallographic transitions [56], decomposition [57,58] and most commonly, nucleation and growth [59,60](*Equation 4*).

(4) $$\alpha =a(1-e^{-{\left(k(t-t_{0})\right)}^{n}})$$

Thte parameter *α *is the extend of reaction, *a *is a fitting parameter that does not deviate much from 1, *k *is the rate constant for reaction, *t *is the reaction time, *t*_*o *_is the induction time, and *n *is a constant that indicates reaction mechanism. Fits were performed in which *n *was set equal to 1 because the scatter in the raw data gives *n *values that are highly variable and inconsistent [9]. Instead of using *n *values to assign reaction pathways, we rely on water chemistry and spectroscopy data to constrain the reaction mechanism to two possibilities.

Growth of semi-crystalline tobermorite was quantified by fitting the time dependent data to the Avrami equation. We calculate a rate constant of *k *= 0.6 (± 0.1) × 10^-5 ^s^-1 ^for the 10:1 batch mixture. For the 5:1 batch mixture, too few time points were collected during the early stages of reaction to reliably calculate a rate constant. However, a rough fit to the experimental data using the Avrami model allow us to estimate that reaction rates are more than an order of magnitude faster than the 10:1 batch reaction (*k *= 1.6 (± 0.8) × 10^-4 ^s^-1^). Figure 13b shows the best fit line to the data (black) and lines that represent the error associated with the fit (red: ± 0.4 × 10^-4 ^s^-1^; blue: ± 0.8 × 10^-4 ^s^-1^). Comparison of our rate data for the 5:1 batch mixture to those reported by Shaw et al [9] show that our rate constant is remarkably close. We calculate *k *~1.2 × 10^-4 ^s^-1 ^for tobermorite growth by extrapolating reaction rates reported by Shaw to our experimental temperature of 150°C (*E*_*a *_= 33 kJ mol^-1^, 15% Al composition; solution:solid = 5; T = 205–310°C, Ref [9]). Interestingly, Shaw used pre-prepared Al-CSH as the starting material instead of a mixture of CaO/SiO_2_/Al_2_O_3 _which suggests that dissolution of CaO/SiO_2 _starting materials and the precipitation of CSH are not rate-limiting. We note that our gel may have varying amounts of Al during the first stages of growth due to slow dissolution of Al_2_O_3 _starting material (^27^Al MAS NMR; Figure 8). Although, Shaw et al reported that growth rates increase with increasing Al concentrations, this affect on reaction rates appears to be minor at temperatures less than 210°C (See Figure 10, Shaw et al, 2000). A rough comparison of the 10:1 and 5:1 rate data show that solution volume has a larger affect on reaction rates than Al content. By reducing the solution volume by half, reaction rates increase by an order of magnitude or more. This suggests that rates are dependent on the solution saturation and that the Gibbs free energy is the reaction driver. We discuss this possibility and another reaction mechanism in the following section.

### Reaction pathways

We consider both heterogeneous nucleation and internal restructuring as possible reaction pathways by coupling diffraction and spectroscopy with solution chemistry data. We collected water chemistry from the 10:1 batch reaction over the course of the reaction, which is shown in Table 2. The 5:1 batch experiments produced too little fluid to sample for solution chemistry analysis. Solution compositions were used to calculate saturation indexes (*SI *= log *Q/K*) for Al-CSH and tobermorite in order to determine differences in interfacial energies (ΔG* = *πσ*^3^*υ*^2^/(*3(k*_*b*_*T ln(SI))*^2^); where *σ *is the interfacial energy). The solubility constant for tobermorite was determined from the water composition for t = 4.5–8 d samples, where the XRD data suggests that reaction is complete and the solution is fully saturated with respect to tobermorite (Figure 12). The solubility constant for Al-CSH was estimated from the water composition at t = 15–24 h (Figure 12), where we assume Al-CSH has reached maximum growth and is fully saturated with respect to the gel phase. It is important to note that the saturation indexes are highly dependent on the equilibrium constant, which is the reason we choose to average several data points that represent maximum growth. The activity coefficients were determined from the calculated solution speciation and the stoichiometry is based on XRF analysis. Stoichiometric data for the CSH solid was collected from a t = 21 h reacted sample. We note that the composition of the CSH gel likely changes during the first 24 h of growth, making it difficult to accurately calculate saturation levels at early time points. This was previously suggested by Thomas et al [61], who found that measuring the solubility of CSH gel presents a challenge due to the continuous range of compositions during hydration. Shown below is the chemical reaction used to model tobermorite (*Equation 5*). Also shown is the chemical equation used to model Al-CSH (*Equation 6*), in which we assume there are no structural hydroxyls. Since only the stoichiometry for the aqueous metal ions and hydrogen ions are required for calculation, this assumption has no bearing on the calculated saturation indexes.

(5) Ca_4.3_Na_1.4_Si_5.3_Al_0.9_O_16_(OH)_2_•4.9H_2_O + 12.7H^+ ^→ 4.3Ca^2+ ^+ 1.4Na^+ ^+ 5.3Sio_2 _(aq) + 0.9Al^3+ ^+ 12.3H_2_O

(6) Ca_3.3_Na_2.1_Si_5.1_Al_1.1_O_16_(OH)_2_•6.2H_2_O + 12.0H^+ ^→ 3.3Ca^2+ ^+ 2.1Na^+ ^+ 5.1Sio_2 _(aq) + 1.1Al^3+ ^+12.2H_2_O

**Figure 14:**
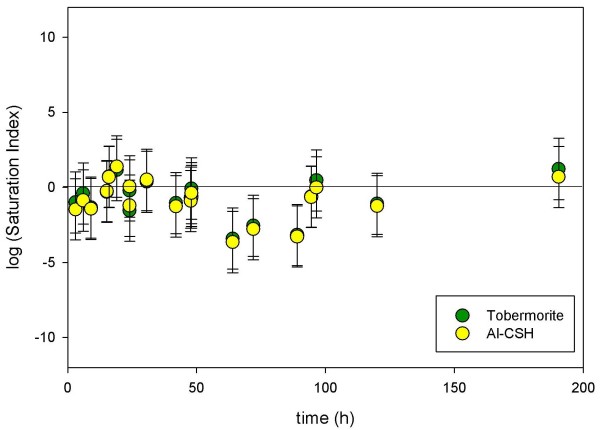
**Solution saturation for tobermorite and Al-CSH as a function of reaction time**. The data show that saturation levels for both solids are similar and that the solution is near equilibrium. All calculations were performed at T = 150°C.

Figure 14 shows that saturation levels for both solids are similar and that the solution is near equilibrium during the course of the reaction. The log *Q/K *values for Al-CSH and tobermorite differ by only one log unit and are within the measured uncertainty. Since only small differences in the saturation conditions are observed, we suggest that (1) either the interfacial energies of meta-stable Al-CSH and tobermorite are similar suggesting that the Gibbs free energy is minimal and is not driving the reaction or (2) that the solution sampled from the top of the solid cake reflects tobermorite solubility and not the combined solubility of the two layered phases.

We consider that the interfacial energies of the two solid phases are similar and that internal restructuring occurs as an alternative mechanism to nucleation and growth. According to the *Ostwald Step Rule*, nucleation of a stable mineral from a solid precursor will occur if the meta-stable assemblage has a lower mineral-solution interfacial energy [62-64]. Once nucleation occurs at the meta-stable phase, cannibalism of the precursor solid allows the stable assemblage to increase in surface area and control the solution composition. Because our data show similar solubilities, nucleation at the expense of the meta-stable phase does not appear to be energetically favorable, resulting in a net thermodynamic driving force that is small. Additionally, we find structural similarities among the two phases. Stoichiometries from XRF indicate similar chemical compositions and NMR data show that both Al-CSH and tobermorite phases contain ^[4]^Al sites and Si-O-Si and Si-O-Al bridging bonds (Figures 4 and 8; short and long reaction times). Cong and Kirkpatrick [18,19] suggest that CSH has a structure in which CSH contains repeating silicate chains and calcium polyhedra similar to tobermorite but with missing chain segments and silicate sites resulting in little long range periodicity. In fact, Shaw et al [9] suggested that CSH is a precursor to tobermorite growth and proposed a phase transformation mechanism in which CaO layers and silicate chains become more ordered through polymerization reactions. Furthermore, there have been several reports of internal restructuring among mineral systems, such as the growth of hematite from ferrihydrite [65,66], the growth of iron-oxyhydroxide biominerals [67], growth and aggregation of TiO_2_solids [68] and the growth of pyrite from a precursor mineral, greigite [69]. Based on our results and those from previous studies, this suggests that little structural rearrangement would be necessary for transformation of Al-CSH to tobermorite.

However, we must consider the possibility that differences in solution saturation conditions could not be identified because the reactive pore water was not sampled in the Al-CSH layer. Due to the limitations of the static batch system, tobermorite formed at the top of the solid cake, where the bulk water was being sampled for analysis. Physical observations of the solid cake point to dissolution and precipitation rather than internal restructuring because the thickness of the tobermorite layer increased over time. Had restructuring taken place, we would expect crystalline and gel domains to be distributed randomly throughout the solid and not separated into two distinct layers. Additionally, we find that reaction rates depend on the solution volume. By reducing the solution volume by half, reaction rates increase by an order of magnitude, suggesting that rates are dependent on solution saturation conditions. We also find from XRD data that growth of tobermorite occurs simultaneously as Al-CSH growth but at a slower rate, indicating that dissolution of the gel dictates the rate at which tobermorite forms. Thus, this data indicates that growth depends on the solution saturation state and that heterogeneous nucleation acts as the thermodynamic driver for growth. However, ΔG* can not be quantified because we did not sample the pore water chemistry within Al-CSH, which is difficult to sample directly.

## Conclusion

Our results show that reaction of CaO/SiO_2_/Al_2_O_3 _in alkaline solution results in three main reaction pathways 1) formation of amorphous and semi-crystalline CSH 2) growth of semi-crystalline tobermorite and 3) re-crystallization of the tobermorite solid. For tobermorite growth, we consider heterogeneous nucleation and internal restructuring as possible mechanistic pathways. We find that bulk rates for tobermorite growth are faster when the solution volume is reduced by half, suggesting that rates are dependent on the solution saturation and that the Gibbs free energy is the reaction driver. However, calculated saturation indexes for Al-CSH and tobermorite differ by less than one log unit and are within the measured uncertainty. We suspect that the solution data most likely reflects tobermorite solubility and not the combined solubility of both Al-CSH and tobermorite phases because the reactive pore water was not sampled in the Al-CSH layer. Although we are not able to resolve the reaction mechanism, our study provides molecular structure and fundamental rate data on cement minerals expected to be present in well-bore systems. Identifying the composition of cement phases and their relative reactivities at experimental temperatures relevant to well-bores is critical towards understanding the long-term fate and storage of CO_2_.

## Competing interests

The authors declare that they have no competing interests.

## Authors' contributions

JRH performed the batch experiments, XRD and NMR data collection, solution speciation calculations, and drafted the manuscript. RSM and SAC conceived of the study and participated in the design and coordination of the study. All authors read and approved the final manuscript.

## Disclaimer

This document was prepared as an account of work sponsored by an agency of the United States government. Neither the United States government nor Lawrence Livermore National Security, LLC, nor any of their employees makes any warranty, expressed or implied, or assumes any legal liability or responsibility for the accuracy, completeness, or usefulness of any information, apparatus, product, or process disclosed, or represents that its use would not infringe privately owned rights. Reference herein to any specific commercial product, process, or service by trade name, trademark, manufacturer, or otherwise does not necessarily constitute or imply its endorsement, recommendation, or favoring by the United States government or Lawrence Livermore National Security, LLC. The views and opinions of authors expressed herein do not necessarily state or reflect those of the United States government or Lawrence Livermore National Security, LLC, and shall not be used for advertising or product endorsement purposes.
